# *FBXL7* Upregulation Predicts a Poor Prognosis and Associates with a Possible Mechanism for Paclitaxel Resistance in Ovarian Cancer

**DOI:** 10.3390/jcm7100330

**Published:** 2018-10-06

**Authors:** Hui-Wen Chiu, Jeng-Shou Chang, Hui-Yu Lin, Hsun-Hua Lee, Chia-Hao Kuei, Che-Hsuan Lin, Huei-Mei Huang, Yuan-Feng Lin

**Affiliations:** 1 Graduate Institute of Clinical Medicine, College of Medicine, Taipei Medical University, Taipei 110, Taiwan; leu3@tmu.edu.tw (H.-W.C.); candycarol0227@gmail.com (H.-Y.L.); kaorulei@yahoo.com.tw (H.-H.L.); pplay1028@gmail.com (C.-H.K.); 2 Division of Nephrology, Department of Internal Medicine, Shuang Ho Hospital, Taipei Medical University, New Taipei City 235, Taiwan; 3 Department of Gastroenterology and Hepatology, Chang Gung Memorial Hospital, Linkou, Taoyuan 333, Taiwan; westlife828@gmail.com; 4 Department of Breast Surgery and General Surgery, Division of Surgery, Cardinal Tien Hospital, Xindian District, New Taipei City 231, Taiwan; 5 Department of Neurology, Shuang Ho Hospital, Taipei Medical University, New Taipei 235, Taiwan; 6 Department of Neurology, School of Medicine, College of Medicine, Taipei Medical University, Taipei 110, Taiwan; 7 Department of Neurology, Vertigo and Balance Impairment Center, Shuang Ho Hospital, Taipei Medical University, New Taipei 235, Taiwan; 8 Taipei Neuroscience Institute, Taipei Medical University, New Taipei City 235, Taiwan; 9 Department of Urology, Division of Surgery, Cardinal Tien Hospital, Xindian District, New Taipei City 231, Taiwan; 10 Graduate Institute of Medical Sciences, College of Medicine, Taipei Medical University, Taipei 110, Taiwan; cloudfrank@gmail.com; 11 Department of Otolaryngology, Taipei Medical University Hospital, Taipei Medical University, Taipei 110, Taiwan

**Keywords:** *FBXL7*, paclitaxel, ovarian cancer, biomarker, in silico analysis

## Abstract

Paclitaxel (PTX) is a common regimen used to treat patients with ovarian cancer. Although approximately 60% of ovarian cancer patients exhibit a pathologic complete response (pCR), approximately 40% of patients appear to be insensitive to PTX adjuvant therapy. Thus, identifying a useful biomarker to predict pCR would be of great help to ovarian cancer patients who decide to receive PTX treatment. We found that *FBXL7* was downregulated in OVSAHO (PTX-sensitive) but upregulated in KURAMOCHI (PTX-resistant) cells after PTX treatment at cytotoxic concentrations. Moreover, our data showed that the fold change of *FBXL7* expression post-treatment with PTX was causally correlated with the 50% inhibitory concentrations (IC_50_) of PTX in a panel of ovarian cancer cell lines. In assessments of progression-free survival probability, high levels of *FBXL7* transcript strongly predicted a poor prognosis and unfavorable response to PTX-based chemotherapy in patients with ovarian cancer. The knockdown of *FBXL7* predominantly enhanced the cytotoxic effectiveness of PTX on the PTX-resistant KURAMOCHI cells. *FBXL7* may be a useful biomarker for predicting complete pathologic response in ovarian cancer patients who decide to receive post-operative PTX therapy.

## 1. Introduction

Ovarian cancer is one of the most common cancer in women globally [1]. Most ovarian cancer patients initially respond to chemotherapy but the majority of patients relapse with a recurrence of the disease which ultimately becomes resistant to chemotherapy [2]. The combination of carboplatin/cisplatin with paclitaxel (PTX) or other chemotherapy agents is the first line of therapeutic strategy in ovarian cancer. However, treatment with various drugs results in a lack of response to therapy in approximately 20% of the patients [3]. Previous studies have demonstrated that patients with drug resistance reflected an inherent mechanism against drug-induced cell death [4]. Several molecular markers, including PI3K/Akt, NF-κB, inhibitors of apoptosis (IAPs), and Bcl-2 family proteins, can drive drug resistance and are associated with resistance to chemotherapy-induced cell death [5,6]. Kamran et al. indicated that Aurora kinase A regulates survivin (a member of human IAPs) stability through targeting F-box and leucine-rich repeat protein 7 (FBXL7) in cancer drug resistance and prognosis [7]. FBXL7 targets Aurora A for polyubiquitylation and proteasomal degradation leading to mitotic cell cycle arrest [8]. Evidence indicates that *FBXL7* is associated with an ovarian cancer gene [9]. The biological role of FBXL7 is not well understood, but F-box proteins constitute one of the subunits of E3 ubiquitin protein ligases involved in phosphorylation-dependent ubiquitination of proteins [10]. The ubiquitin-proteasome system (UPS) is a major degradation system for short-lived proteins. The ubiquitination of a target protein in humans is orchestrated by an enzymatic cascade including ubiquitin-activating enzyme (E1), ubiquitin-conjugating enzymes (E2), and ubiquitin E3 ligase of which hundreds exist in cells [11]. Previous studies have demonstrated that among these E3 ligases, the SCF superfamily modulates diverse biological processes [12]. The SCF complex is composed of a catalytic core containing Skp1, Cul1, Rbx1, and an F-box protein [13]. Furthermore, F-box proteins play a key role in cell growth and differentiation, signal transduction, survival, and apoptosis [8,14]. Although these data suggest a role for FBXL7 in controlling cell proliferative activity and viability, further studies investigating the relationship between *FBXL7* and drug resistance in ovarian cancer are needed.

Despite significant advances in the treatment of cancer, drug resistance remains a major clinical barrier to successful treatment and leads to poor prognosis for the patients [15,16]. In this study, we found that *FBXL7* could serve as a poor prognosis marker in ovarian cancer patients. *FBXL7* upregulation is associated with poor progression-free survival (PFS) rates. Furthermore, *FBXL7* upregulation is positively correlated with IC_50_ concentrations of PTX in ovarian cancer cell lines. These results indicated that *FBXL7* plays a crucial role in PTX-resistant ovarian cancer cells. 

## 2. Experimental Section

### 2.1. Cell Culture

KURAMOCHI cell line was obtained from the Japanese Collection of Research Bioresources Cell Bank (JCRB) and a gift of Dr. Michael Hsiao from Genomics Research Center at Academia Sinica in Taiwan. The cells were cultured in RPMI-1640 medium (Invitrogen, Carlsbad, CA, USA) with 10% fetal calf serum (FCS) and incubated at 37 °C with 5% CO_2_. Cells were routinely authenticated on the basis of short tandem repeat (STR) analysis, morphologic and growth characteristics, and mycoplasma detection.

### 2.2. Reverse Transcriptase-Polymerase Chain Reaction (RT-PCR)

Total RNA was extracted from cells using TRIzol extraction kit (Invitrogen). Aliquots (5 μg) of total RNA were treated with M-MLV reverse transcriptase (Invitrogen) and then amplified with Taq-polymerase (Protech, Hong Kong, China) using paired primers (for FBXL7, forward-CACGCAGCTCACCCACCTCTA and reverse-GGTGCAGTTCTTGGCGAGGT; for glyceraldehyde-3-phosphate dehydrogenase (GA PDH), forward-AGGTCGGAGTCAACGGATTTG and reverse-GTGATGGCATGGACTGTGGTC).

### 2.3. Cell Tranfection

Non-silencing and FBXL7 shRNA (target sequence: sh1-GCATCTCATCTGACGTGAGTT; sh2-CCACCGAATCTCCCAGGATTT)-containing lentiviral particles were obtained from National RNAi Core Facility Platform in Taiwan and transiently transfected to KURAMOCHI cells in a polybrene (10 μg/mL, Santa Cruz Biotechnology, Inc., Santa Cruz, CA, USA)-containing medium for 24 h prior to paclitaxel (Sigma-Aldrich, St. Louis, MO, USA) treatment for another 48 h. 

### 2.4. MTT Assay

Cells (1 × 10^5^ /mL) were seeded into a 96-well culture plate. After the incubation with paclitaxel for 48 h, 10 μL of MTT (3-(4,5-dimethylthiazol-2-yl)-2,5-diphenyltetrazolium bromide) (Molecular Probe, Invitrogen) stock solution was added into each well. The conversion of MTT to formazan by viable cells was performed at 37 °C for another 4 h. After the reaction, 100 μL of DMSO solution was added into each well in order to solubilize the formazan precipitates. The levels of formazan were determined by optical density at 540 nm using an Enzyme-linked immunosorbent assay (ELISA) (Molecular Devices, San Jose, CA, USA) reader for calculating cell survival rates.

### 2.5. Microarray and RNA Sequencing Data Processing 

Microarray results with accession numbers GSE50831 and the related clinical data were obtained from the Gene Expression Omnibus (GEO) database on the NCBI website. Affymetrix DAT files were processed using the Affymetrix Gene Chip Operating System (GCOS) to generate .CEL files. Raw intensities in the .CEL files were normalized by robust multi-chip analysis (RMA), and fold-change analysis was done using GeneSpring GX11 (Agilent Technologies, Santa Clara, CA, USA). Relative mRNA expression levels were normalized by the median of all samples and presented as log_2_ values. The processed data of microarray and RNA sequencing (RNA-seq) for the *FBXL7* gene and the clinicopathological information of patients with ovarian serous cystadenocarcinoma deposited in The Cancer Genome Atlas (TCGA) database were downloaded from the Cancer Browser website. 

### 2.6. Immunohistochemistry Staining Analysis

The paraffin-embedded tissue microarray of ovarian cancers purchased from SuperBioChips (Table S1) were heat and deparaffinized using xylene, and rehydrated in a graded series of ethanol with a final wash in tap water. Antigen retrieval was treated with Target Retrieval Solution (DAKO) in a Decloaking Chamber (Biocare Medical, Pacheco, CA, USA). Endogenous peroxidase activity was quenched by hydrogen peroxide. Sections were then incubated with anti-FBXL7 antibody (Aviva System Biology, San Diego, CA, USA, Catalog#: ARP43132) at 4 °C overnight. A Vectastain ABC peroxidase system (Vector Laboratories, Burlingame, CA, USA) was used to detect the reaction products.

### 2.7. Kaplan-Meier Analyses

The SurvExpress, K-M Plotter, and TCGA databases containing records of ovarian cancer patients with follow-up time intervals were used to estimate the prognostic significance of *DGKH*, *FBXL7,* and *MFSD6* transcripts under the condition of progression or recurrence-free survival probability using Kaplan-Meier analysis. Moreover, the 614 and 1435 ovarian cancer patients without any treatment and the 381 and 715 ovarian cancer patients receiving post-operative chemotherapy from the K-M Plotter database were recruited to perform another Kaplan-Meier analysis for *DGKH*, *FBXL7*, and *MFSD6* transcripts under the condition of PFS probability.

### 2.8. Univariate and Multivariate Analyses

The 578 ovarian serous cystadenocarcinoma patients from the TCGA database were used to perform univariate and multivariate analyses using a Cox regression test. *FBXL7* expression levels and clinical data including age, pathological stage, and histologic grade were input as variables in the Cox regression test using recurrence-free survival condition. 

### 2.9. In Silico Analysis

Genes with a 1.5-fold-change threshold relative to control cells in OVSAHO and KURAMOCHI cells post-treatment with PTX at 10× IC_50_ concentrations (Table S2) were uploaded to the Ingenuity Pathway Analysis (IPA) website (Ingenuity Systems, www.ingenuity.com). Data from computational predictions for the activation or inhibition status of upstream regulators were then output as a text file (Table S3). Consensus upstream regulators with significant (*p* < 0.05) z-scores from the in silico analysis of PTX-treated OVSAHO and KURAMOCHI cells were analyzed in a PivotTable report and plotted as a dotplot in Microsoft Excel. 

### 2.10. Statistical Analyses

SPSS 17.0 software (Informer Technologies, Roseau, Dominica) was used to analyze statistical significance. Pearson’s test was performed to estimate the association among *DGKH*, *FBXL7*, and *MFSD6* mRNA and PTX IC_50_ concentrations in the panel of breast cell lines. Pearson’s test was also used to evaluate the statistical significance among the transcriptional profiling of *DGKH*, *FBXL7*, and *MFSD6* in 568 ovarian serous cystadenocarcinoma patients from the TCGA database. Survival probabilities were determined by Kaplan-Meier analysis and log-rank tests. One-way ANOVA with Tukey’s test was used to estimate the differences in mRNA levels of *DGKH*, *FBXL7*, and *MFSD6* in D OVSAHO and KURAMOCHI cells after PTX treatment. Mann-Whitney U tests were used to analyze non-parametric data. *p* values < 0.05 in all analyses were considered statistically significant.

## 3. Results

### 3.1. Upregulation of DGKH, FBXL7 and MFSD6 Is Involved in the Mechanism Underlying PTX Resistance 

**Figure 1:**
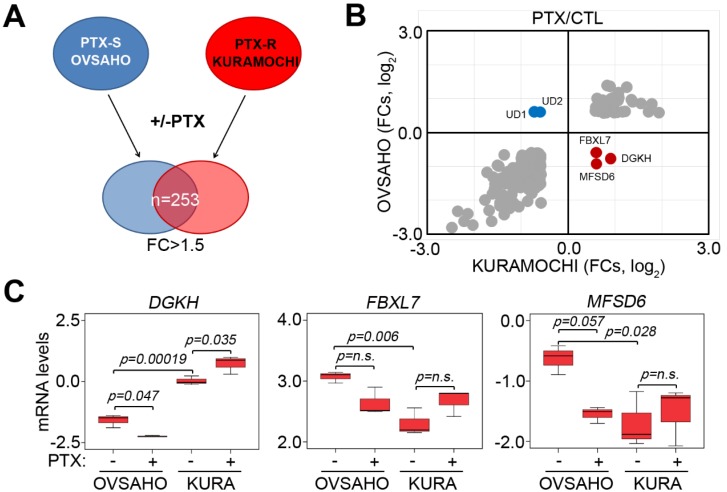
Upregulation of *DGKH*, *FBXL7*, and *MFSD6* involved in the mechanism underlying Paclitaxel (PTX) resistance in PTX-insensitive ovarian cancer cells. (**A**) A flowchart for identifying consensus genes with 1.5-fold change (FC) posttreatment with PTX at the concentration of 10 × IC_50_ for 24 h in PTX-sensitive (PTX-S) OVSAHO cells and PTX-resistant (PTX-R) KURAMOCHI cells. (**B**) The dotplot for the mRNA levels (log_2_) of 253 consensus genes identified as the strategy shown in (**A**). (**C**) The mRNA levels of *DGKH*, *FBXL7* and *MFSD6* in OVSAHO and KURAMOCHI cells post-treatment without or with PTX at the concentration of 10 × IC_50_ for 24 h. Data from three independent experiments are shown as the median ± SD. The significant differences were analyzed by one-way ANOVA using Turkey’s test.

By performing the in silico analysis against the microarray dataset GSE50831, we identified 253 consensus genes with 1.5-fold changes (FC) post-treatment with PTX at the concentration of 10× IC_50_ for 24 h in PTX-sensitive OVSAHO cells and PTX-resistant KURAMOCHI cells (Figure 1A). Among 253 consensus genes, the mRNA levels of *DGKH*, *FBXL7*, and *MFSD6* were predominantly downregulated in OVSAHO cells but upregulated in KURAMOCHI cells after the treatment with PTX (Figure 1B,C). Conversely, UD1 and UD2 mRNA levels were increased in OVSAHO cells but decreased in KURAMOCHI cells post-treatment with PTX. Furthermore, *DGKH* and *MFSD6* mRNA levels detected by different probes yielded similar results (Figure S1).

**Figure 2:**
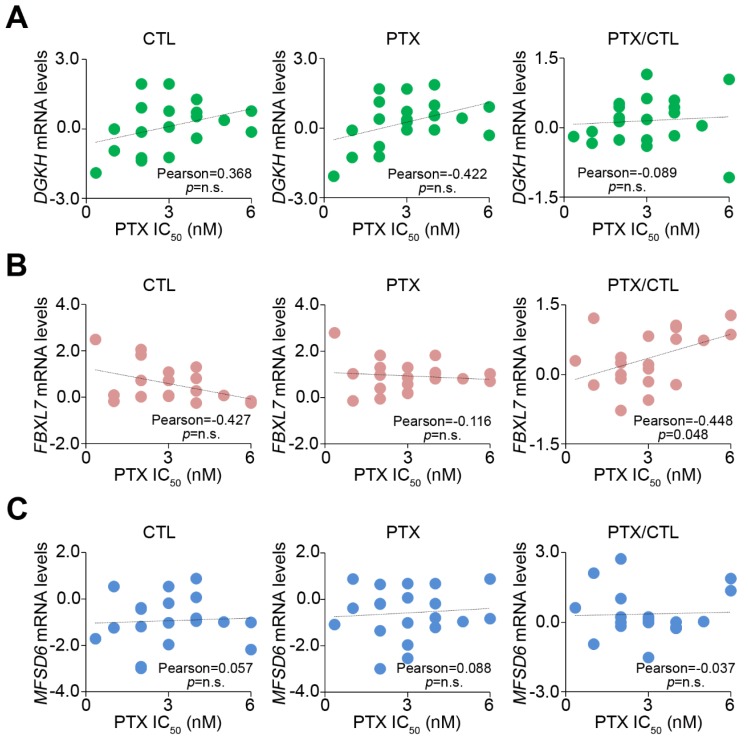
The enhanced expression of *FBXL7* positively correlates with PTX IC_50_ concentrations in a panel of ovarian cancer cell lines. (**A**–**C**) Correlations among *DGKH*, *FBXL7* and *MFSD6* mRNA level and PTX IC_50_ concentration in the tested ovarian cancer cell lines. The statistical significance of correlations was analyzed by using Pearson’s test. The symbol “n.s.” denotes not significant. Each dot in the dotplot indicates the median of mRNA levels from three independent experiments.

We further investigated the correlation between the IC_50_ concentrations of PTX and the mRNA levels of *DGKH*, *FBXL7* and *MFSD6* in a panel of ovarian cell lines; A2780, CaOv3, COLO-720, COLO-704, COV362, COV434, COV504, COV644, EFO-21, EFO-27, KURAMOCHI, OV56, OV-90, OVCAR-3, OVCAR-4, OVISE, OVSAHO, OVTOKO, SK-OV-3, TOV-112D, and TOV-21G treated with or without PTX at their respective 10-fold IC_50_ concentrations (Figure 2). The fold change of *FBXL7* transcript post-treatment with PTX was shown to be positive, whereas the *DGKH* and *MFSD6* mRNA levels did not appear to be significantly correlated with the PTX IC_50_ concentrations of each tested cell line. Therefore, *FBXL7* upregulation might play a key role in the mechanism for PTX-resistance in ovarian cancer cells.

### 3.2. FBXL7 Upregulation Is Associated with Poor Progression-Free Survival (PFS) Rates in Ovarian Cancer Patients

**Figure 3:**
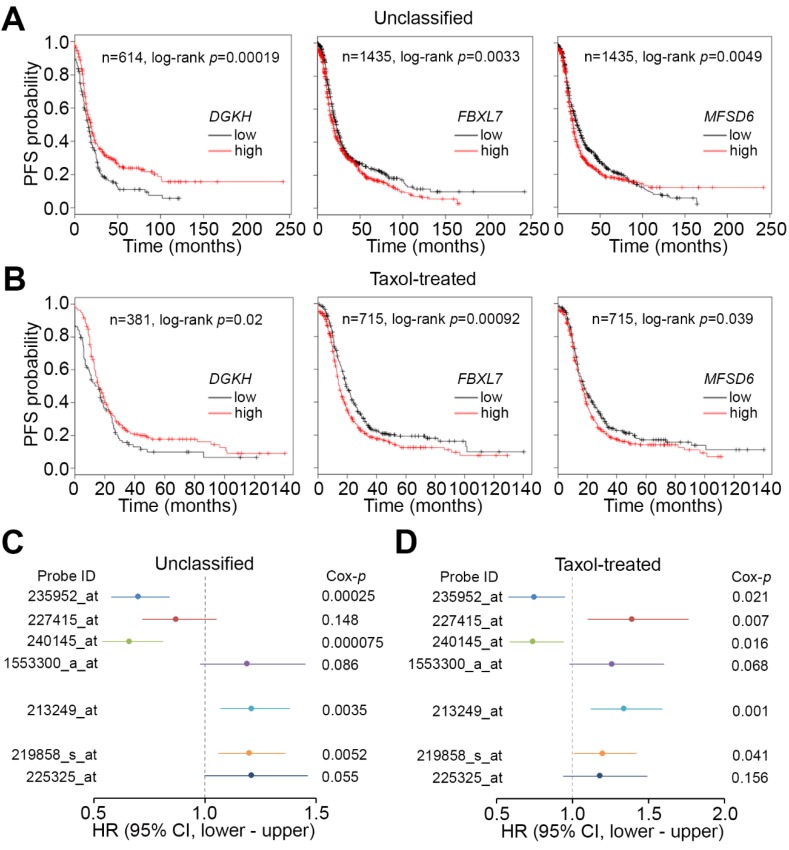
*FBXL7* upregulation refers to a poor progression-free survival (PFS) rates in ovarian cancer patients. (**A** and **B**) K-M analysis for *DGKH*, *FBXL7*, and *MFSD6* mRNA expression under the condition of PFS probability in ovarian cancer patients who are unclassified (**A**) or classified into the taxol-treated cohort (**B**) using the K-M Plotter database. (**C** and **D**) Cox regression test using univariate model against different probe identifiers (ID) for *DGKH* (235952_at, 227415_at, 240145_at and 1553300_a_at), *FBXL7* (213249_at), and *MFSD6* (219858_s_at and 225325_at) in a microarray analysis using the K-M Plotter database under the condition of PFS probability. HR denotes hazard ratio.

To estimate the clinical relevance of *DGKH*, *FBXL7*, and *MFSD6* in patients with ovarian cancer, we next performed Kaplan-Meier analysis using the K-M Plotter database. Under the condition of progression-free survival (PFS) probability, we found that *FBXL7* and *MFSD6* transcripts at high levels—but *DGKH* at low levels—significantly (*p* < 0.05) predicted a poor prognosis in the unclassified ovarian cancer patients (Figure 3A). Similar views were also found in the ovarian cancer patients with post-operative taxol adjuvant therapy (Figure 3B). In addition, Kaplan-Meier analysis for other *DGKH* and *MFSD6* microarray probes using the K-M Plotter database against the unclassified ovarian cancer patients yielded similar results under the condition of PFS probability (Figure S2). In the ovarian cancer patients with taxol adjuvant therapy, the other *DGKH*, except probes 227415_at and 1553300_a_at, and *MFSD6* microarray probes displayed similar prognostic significance under PFS probability (Figure S3). Next, we performed Cox regression tests using a univariate model against different probe identifiers for *DGKH*, *FBXL7* and *MFSD6* in the microarray analysis using the K-M Plotter database under the condition of PFS probability. The data showed that the high-level transcripts determined by probe identifiers for *DGKH* (except probes 235952_at, 227415_at and 240145_at), *FBXL7*, and *MFSD6* were associated with an unfavorable hazard ratio in the unclassified cohort (Figure 3C). Similarly, in the ovarian cancer patients receiving taxol adjuvant therapy, the elevated mRNA levels detected by probes for *DGKH* (except probes 235952_at and 240145_at), *FBXL7*, and *MFSD6* appeared to be correlated with poor outcomes (Figure 3D). Since *FBXL7* showed the strongest significance in both Kaplan-Meier analysis and Cox regression tests, we focused on the investigation of its role in predicting and mediating PTX resistance in ovarian cancer. 

**Figure 4:**
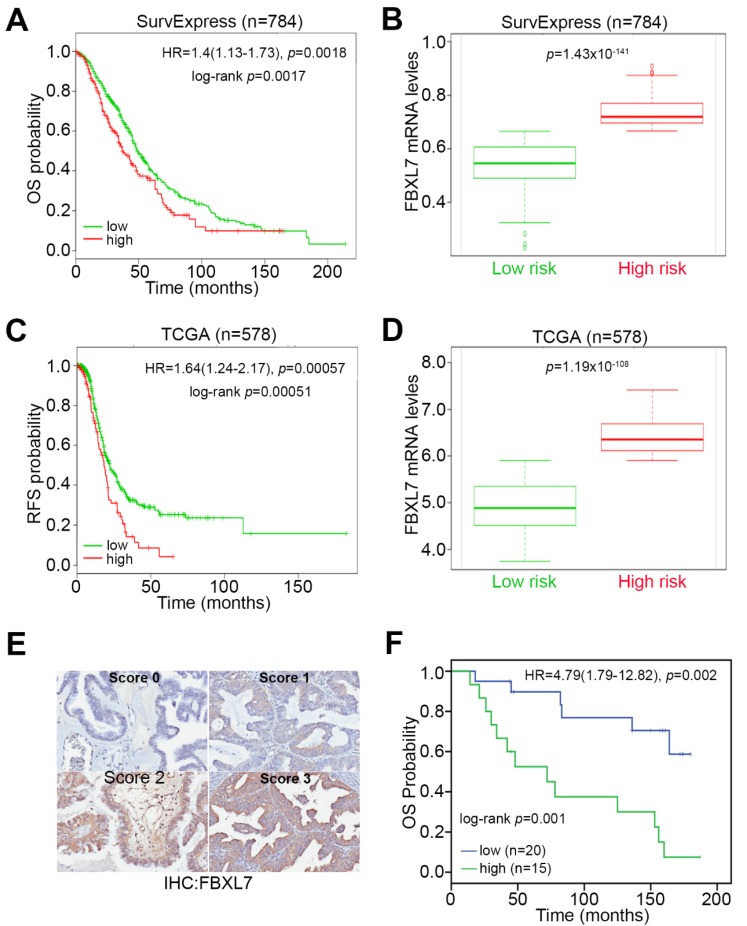
*FBXL7* upregulation refers to an unfavorable outcome in ovarian cancer patients. (**A**) K-M analysis for *FBXL7* expression under the condition of overall survival (OS) probability in ovarian cancer patients using the SurvExpress database. (**B**) Boxplot for the mRNA levels of *FBXL7* in low (green) and high (red)-risk cohorts in (**A**). (**C**) K-M analysis for *FBXL7* expression under the condition of recurrence-free survival (RFS) probability in ovarian cancer patients using the TCGA database. (**D**) Boxplot for the mRNA levels of *FBXL7* in low (green) and high (red)-risk cohorts in (**C**). (**E** and **F**) Immunohistochemistry (IHC) staining for FBXL7 protein (**E**) in ovarian cancer tissues and Kaplan-Meier analysis (**F**) under the condition of OS probability basing on the IHC staining intensities (low, Scores 0 and 1; high, Scores 2 and 3) of FBXL7 against the ovarian cancer patients with papillary serous cystadenocarcinoma. In **A**, **C**, **F**, HR denotes hazard ratio.

To further validate the prognostic significance of *FBXL7* transcripts, we performed another Kaplan-Meier analysis using the SurvExpress database. Under the condition of overall survival (OS), high levels of *FBXL7* transcripts were significantly (*p* = 0.0017) associated with a poor survival rate and unfavorable hazard ratio (HR = 1.4, *p* = 0.0018) in ovarian cancer patients (Figure 4A,B). Furthermore, we utilized the TCGA database of patients with ovarian serous cystadenocarcinoma to estimate the risk of cancer recurrence using the condition of recurrence-free survival probability, which commonly reflects a complete pathologic response in patients who received cancer therapies. The data revealed that high levels of *FBXL7* transcripts predict an increased risk for cancer recurrence, with a statistical significance at log-rank *p* = 0.00051; HR = 1.64, *p* = 0.00057 (Figure 4C,D). Using this TCGA database, we also performed Cox univariate and multivariate analyses for age, pathologic stages, histologic grades, and *FBXL7* expression in ovarian serous cystadenocarcinoma patients. The data indicated that high levels of *FBXL7* transcripts could be an independent factor for predicting an unfavorable risk for cancer recurrence after adjuvant chemotherapy in ovarian cancer patients (Table 1). Besides, we also performed immunohistochemistry (IHC) staining (Figure 4E) against a tissue microarray composed of various ovarian cancer types (Table S1) to estimate the prognostic significance of FBXL7 protein levels in ovarian cancer patients. The IHC results revealed that high protein levels of FBXL7 refers to a poor overall survival rate and an increased hazard ratio in the enrolled ovarian cancer patients (Figure 4F). 

### 3.3. Possible Mechanism Underlying PTX Resistance in Ovarian Cancer Cells

**Figure 5:**
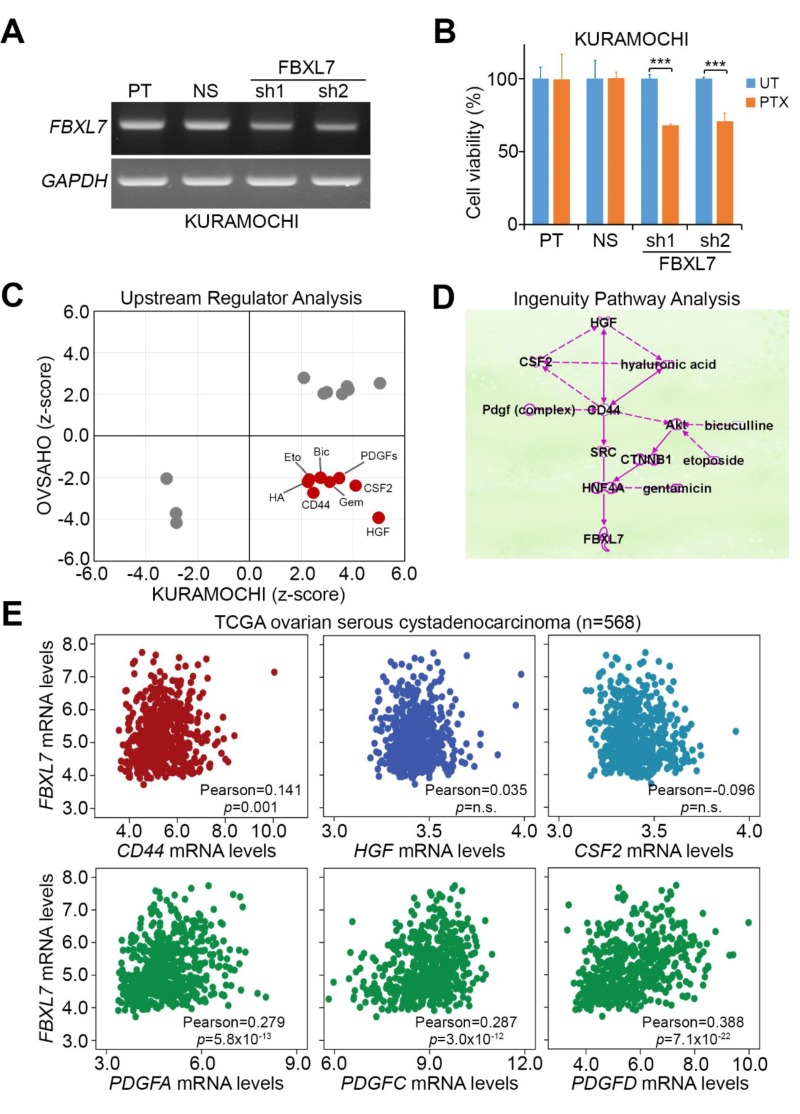
Possible mechanism underlying PTX resistance in ovarian cancer cells. (**A**) The mRNA levels of FBXL7 and GAPDH detected by RT-PCR experiment in parental (PT) KURAMOCHI cells transfected without or with non-silencing (NS) or 2 independent FBXL7 shRNA (sh) clones for 24 h. GAPDH was used as an internal control of the experiment; (**B**) Cell viability measured by MTT assay in parental, non-silencing or FBXL7-silencing KURAMOCHI cells treated with PTX at 10 × IC_50_ concentration (60 nM). The symbol (***) denotes statistical significance at *p* < 0.001 in the Mann–Whitney U test; (**C**) The in silico analysis of consensus upstream regulators that are possibly activated or inhibited after PTX treatment at the concentration of 10 × IC_50_ for 24 h in OVSAHO and KURAMOCHI cells using Ingenuity Pathway Analysis (IPA) software; (**D**) Interactions among the identified upstream regulators that are highly predicted to be activated in PTX-treated KURAMOCHI cells with PTX resistance using IPA simulation. Solid and dashed lines represent direct and indirect interactions, respectively, among the tested molecules; (**E**) Correlations among *FBXL7* mRNA levels and the identified upstream regulators *CD44*, *HGF*, *CSF2*, *PDGFA*, *PDGFC*, and *PDGFD* in patients with ovarian serous cystadenocarcinoma using TCGA database. The statistical significance of correlations was analyzed by pearson’s test.

To realize a direct association of *FBXL7* expression with PTX resistance, we performed the gene knockdown experiment for *FBXL7* in PTX-resistant KURAMOCHI cells. Compared to parent and non-silencing control cells, artificially silencing *FBXL7* dramatically reduced *FBXL7* transcripts (Figure 5A) and significantly (*p* < 0.001) enhanced the cytotoxic effectiveness of PTX (Figure 5B) on PTX-resistant KURAMOCHI cells. To delineate a possible mechanism in which *FBXL7* upregulation triggers PTX resistance in ovarian cancer, we next performed an in silico computational simulation using Ingenuity Pathway Analysis (IPA) software to identify potentially activated or inhibited upstream regulators that mediate the transcription of *FBXL7*. We found that several consensus upstream regulators are possibly inhibited or activated after PTX treatment in OVSAHO and KURAMOCHI cells, respectively, in the IPA prediction (Figure 5C). By using the IPA software, we also identified the presumable interactions among the identified upstream regulators (Figure 5D). To evaluate which extracellular molecules among those predicted to be activated from the IPA results are potentially able to trigger signaling pathways for elevating *FBXL7* expression, we performed a Pearson’s correlation test to estimate the relationships among *FBXL7*, *CD44*, *HGF*, *CSF2*, *PDGFA*, *PDGFB*, *PDGFC*, and *PDGFD* transcripts in patients with ovarian serous cystadenocarcinoma using the TCGA database. The results showed that *FBXL7* expression was positively correlated with *CD44*, *PDGFA*, *PDGFC*, and *PDGFD* in ovarian serous cystadenocarcinoma tissues with a statistical significance (*p* = 0.001 or *p* < 0.001) (Figure 5E and Figure S4).

## 4. Discussion

UPS is involved in the regulation of a number of processes such as cell cycle control, differentiation, antigen processing, and angiogenesis [17]. The E3 ligases mediate the last step of the ubiquitination pathway. E3 interacts both with E2-Ub and the substrate to be ubiquitinated. Thus E3 determines the selectively of the ubiquitination process [18]. Recent evidence shows that UBR5 (an E3 ubiquitin ligase) knockdown enhances Bax activation and sensitizes resistant cells to cisplatin-induced apoptosis. Furthermore, UBR5 expression was higher in ovarian cancers from cisplatin-resistant patients than from cisplatin-responsive patients [19]. Another recent study concluded that E3 ubiquitin ligase HOIP attenuates apoptotic cell death induced by cisplatin in several cancer cells, including ovarian cancer [20]. FBXL7 is one of the subunits of E3 ubiquitin protein ligases. In the present study, the mRNA levels of *FBXL7* were downregulated in a PTX -sensitive ovarian cancer cell line (OVSAHO) but upregulated in PTX-resistant KURAMOCHI cells treated with PTX (Figure 1B,C and Table S2). Furthermore, *FBXL7* mRNA levels were positively correlated with the PTX IC_50_ concentrations of several ovarian cancer cell lines (Figure 2B). These results showed that a novel gene, *FBXL7*, may control PTX resistance in ovarian cancer cells.

Our current data indicate that high levels of *FBXL7* mRNA indicated a significantly poor PFS compared with low levels of those mRNA in ovarian cancer patients who were in the treated with PTX cohort (Figure 3). Patients with *FBXL7* upregulation had a significantly unfavorable OS compared with those patients with *FBXL7* downregulation in the SurvExpress database and the TCGA database (Figure 4A–D). Cox univariate and multivariate analyses also showed that *FBXL7* upregulation and pathological stage significantly predicted a poor RFS probability after adjuvant chemotherapy in ovarian serous cystadenocarcinoma (Table 1). Furthermore, high protein levels of FBXL7 refers to a poor OS and an increased hazard ratio in the enrolled ovarian cancer patients (Figure 4E,F). However, there are few published articles describing the relationship between *FBXL7* and survival outcome in ovarian cancer. Previous studies have demonstrated that a single nucleotide polymorphism (SNP) in *FBXL7* showed the strongest associations in *BRCA2* mutation carriers of breast cancer [21]. Lifetime risks of ovarian cancer were 54% for *BRCA1* and 23% for *BRCA2* mutation carriers [22]. FBXL7 is also a transcription factor and contributes to cell cycle regulation [23]. Considering the gene transcription regulatory function of the SCF complex as a member, our predicted gene FBXL7 might contribute to the transcription regulation of certain genes physically or psychologically [14]. Recent studies have shown that *FBXL7* expression was positively correlated with *CD44*, *PDGFA*, *PDGFC*, and *PDGFD* in ovarian serous cystadenocarcinoma (Figure 5C and Figure S4). Several published studies implicate PDGF and PDGF-receptor (PDGF-R) in ovarian cancer growth [24,25]. It has been reported that 73% of ovarian carcinomas are PDGF(+) and 36% are PDGF-Rα(+) [26]. In addition, CD44 is a cell-surface glycoprotein involved in cell-cell interactions, cell adhesion, and migration [27,28]. The expression of CD44 correlates with tumor initiation, growth, development of drug resistance, and metastases in ovarian cancer [29,30,31]. These previous studies suggest that PDGF and CD44 may play a role in the biology of ovarian cancer. Although this research was carefully prepared, there were some limitations and shortcomings. For example, the trend lines of the survival curve did not separate much in the Kaplan-Meier analysis for *FBXL7* transcript against ovarian cancer patients derived from K-M Plotter and TCGA public databases, even though the statistical *p* value was low. In contrast, Kaplan-Meier analysis for *FBXL7* protein levels derived from IHC staining against tissue microarray showed more separate trend lines. The majority of ovarian cancer types were serous cystadenocarcinoma (>90%) in the two public databases, whereas tissue microarray was composed of the mixed-type ovarian cancers. Therefore, the determination of *FBXL7* transcript levels in IHC experiment against tissue microarray is still needed to further clarify the correlation between *FBXL7* transcript and protein levels and their clinical relevance. Otherwise, we cannot exclude the interference of enrolled cohort sizes and ovarian cancer types in the prognostic estimation against *FBXL7* transcript as we utilized the public databases. On the other hand, further experimental validations are also needed to prove the interactions among *FBXL7*, *PDGF* and *CD44* in conferring ovarian cancer with PTX resistance, even though the in silico analysis significantly predicted the activations of these signal transducers in the PTX-resistant ovarian cancer cells.

## 5. Conclusions

In summary, *FBXL7* upregulation plays a key role in PTX-resistant response in ovarian cancer cells. A high expression rate of *FBXL7* is associated with poor survival of ovarian cancer patients. Furthermore, the mRNA levels of *FBXL7* in the high-risk cohort were significantly upregulated compared to the low-risk cohort in ovarian cancer patients. *FBXL7* upregulation and pathological stage significantly predicted a poor RFS probability after adjuvant chemotherapy in ovarian cancer. In addition, *FBXL7* expression was positively correlated with *CD44*, *PDGFA*, *PDGFC*, and *PDGFD* in ovarian serous cystadenocarcinoma. 

## Figures and Tables

**Table 1:** Cox univariate and multivariate analyses under the condition of recurrence-free survival probability in association with *FBXL7* mRNA expression levels and pathological stage derived TCGA (The Cancer Genome Atlas) cohort with ovarian serous cystadenocarcinoma.

Recurrence-Free Survival (*n* = 578)				
Variables	Crude HR (95% CI)	*p*	Adjusted HR (95% CI)	*p*
Age				
59<	1	NA	1	NA
59	1.00 (0.79–1.26)	0.987	1.00 (0.79–1.26)	0.987
Pathologic stage				
IA-IIIB	1	NA	1	NA
IIIC-IV	1.76 (1.22–2.53)	0.002	1.69 (1.17–2.45)	0.005
Histologic grade				
G1–G2	1	NA	1	NA
G3–G4	1.30 (0.94–1.79)	0.109	1.25 (0.91–1.74)	0.174
FBXL7 expression				
low	1	NA	1	NA
high	1.52 (1.14–2.04)	0.005	1.51 (1.13–2.02)	0.006

## Supplementary Materials

The following are available online at http://www.mdpi.com/2077-0383/7/10/330/s1, Figure S1: Transcriptional profiling of *DGKH* and *MFSD6* with other probe identifiers in microarray analysis against OVSAHO and KURAMOCHI cells post-treatment without or with PTX, Figure S2: Prognostic significance of different *DGKH* and *MFSD6* probes in K-M Plotter database against ovarian cancer patients, Figure S3: Prognostic significance of different *DGKH* and *MFSD6* probes in K-M Plotter database against ovarian cancer patients who received post-operative taxol-based chemotherapy, Figure S4: Correlation between *FBXL7* and *PDGFB* mRNA levels in tissues derived from patients with ovarian serous cystadenocarcinoma using TCGA database, Table S1: List of consensus genes with 1.5-fold changes in KURAMOCHI and OVSAHO cells following treatment with PTX, Table S2: List of consensus upstream regulators that are computationally predicted to be activated or inhibited after PTX treatment in KURAMOCHI and OVSAHO cells, Table S3: List of consensus upstream regulators that are computationally predicted to be activated or inhibited after paclitaxel treatment in KURAMOCHI and OVSAHO cells.
