# Supplementary material for: FBXL7 Upregulation Predicts a Poor Prognosis and Associates with a Possible Mechanism for Paclitaxel Resistance in Ovarian Cancer
Source: J Clin Med. 2018 Oct 6;7(10):330. doi: 10.3390/jcm7100330 (PMC6209951; doi:10.3390/jcm7100330)
Supplement: Supplementary file 1 [file jcm-07-00330-s001.pdf]

Supplementary Information

Table of contents

1. Table S1. The survival data for ovarian cancer patients enrolled in IHC experiment and Kaplan-Meier analysis.
2. Table S2. List of consensus genes with 1.5-fold changes in KURAMOCHI and OVSAHO cells following treatment with paclitaxel.
3. Table S3. List of consensus upstream regulators that are computationally predicted to be activated or inhibited after paclitaxel treatment in KURAMOCHI and OVSAHO cells.
4. Figure S1. Transcriptional profiling of *DGKH* and *MFSD6* with other probe identifiers in microarray analysis against OVSAHO and KURAMOCHI cells post-treatment without or with paclitaxel.
5. Figure S2. Prognostic significance of different *DGKH* and *MFSD6* probes in K-M Plotter database against ovarian cancer patients.
6. Figure S3. Prognostic significance of different *DGKH* and *MFSD6* probes in K-M Plotter database against ovarian cancer patients who received post-operative taxol-based chemotherapy.
7. Figure S4. Correlation between *FBXL7* and *PDGFB* mRNA levels in tissues derived from patients with ovarian serous cystadenocarcinoma using TCGA database.

Supplementary Table 1. The survival data for ovarian cancer patients enrolled in IHC experiment and Kaplan-Meier analysis.

Patient No.	Organ	Diagnosis	Follow-up result	Follow-up months	FBXL7 low_1, high_2
4	Ovary	serous cystadenocarcinoma	1	125	2
6	Ovary	granulosa cell tumor	0	187	2
7	Ovary	endometrioid carcinoma	1	72	2
8	Ovary	mucinous cystadenoma of borderline malignancy	1	160	2
9	Ovary	serous cystadenofibroma	1	78	2
12	Ovary	papillary serous cystadenocarcinoma	1	153	2
13	Ovary	endometrioid carcinoma	1	83	1
15	Ovary	papillary serous cystadenocarcinoma	1	42	2
16	Ovary	papillary serous cystadenocarcinoma	1	26	2
17	Ovary	struma ovarii	0	180	1
18	Ovary	endometrioid adenoacanthofibroma	1	156	2
20	Ovary	papillary serous cystadenocarcinoma	1	48	2
21	Ovary	undifferentiated carcinoma	1	164	1
22	Ovary	clear cell carcinoma	1	30	2
24	Ovary	common epithelial carcinoma , poorly differentiated	0	174	1
25	Ovary	malignant muellerian mixed tumor	0	174	1
26	Ovary	dysgerminoma	0	174	1
28	Ovary	papillary serous cystadenocarcinoma	0	172	1
29	Ovary	sertoli-leydig cell tumor	1	82	1
32	Ovary	papillary serous cystadenocarcinoma	1	21	2
35	Ovary	granulosa-theca cell tumor	1	136	1
38	Ovary	clear cell carcinoma	0	161	1
39	Ovary	malignant muellerian mixed tumor	1	18	1
40	Ovary	clear cell carcinoma	0	159	1
41	Ovary	embryonal carcinoma	1	14	2
42	Ovary	granulosa cell tumor	0	157	1

43	Ovary	dysgerminoma	0	150	1
44	Ovary	papillary serous cystadenocarcinoma	1	34	2
47	Ovary	adenocarcinoma from salpinx	0	136	1
51	Ovary	serous cystadenocarcinoma, moderately differentiated	1	45	1
53	Ovary	serous cystadenocarcinoma, moderately differentiated	0	47	1
54	Ovary	endometrioid carcinoma	0	47	1
56	Ovary	adenocarcinoma, poorly differentiated	0	45	2
57	Ovary	clear cell carcinoma	0	45	1
59	Ovary	clear cell carcinoma	0	44	1

Supplementary Table 2. List of consensus genes with 1.5-fold changes in KURAMOCHI and OVSAHO cells following treatment with paclitaxel.

Probe ID	KURAMOCHI	OVSAHO	Gene Symbol
202859_x_at	1.928295	0.595535	CXCL8
234989_at	1.783833	0.857776	MIR612
211506_s_at	1.710674	0.617805	CXCL8
225239_at	1.548148	0.870925	MIR612
236841_at	1.373972	0.913855	LOC100134445
240971_x_at	1.341825	0.973964	
213813_x_at	1.243734	1.340563	
220295_x_at	1.214665	0.633918	DEPDC1
209118_s_at	1.154372	0.615633	TUBA1A
237238_at	1.074866	1.037621	WWC1
214041_x_at	1.068836	1.045738	RPL37A
224771_at	1.067367	1.182241	NAV1
235577_at	1.060006	0.951836	ZNF652
235984_at	1.040285	0.608362	
214001_x_at	0.974973	0.91309	RPS10
243981_at	0.974575	0.596066	STK4
236533_at	0.956902	0.783482	ASAP1
224559_at	0.950349	0.69671	MALAT1
211074_at	0.939653	1.029186	
231199_at	0.92521	0.721866	RP11-271C24.3
228613_at	0.918065	0.640564	RAB11FIP3
235757_at	0.913377	0.645474	
231211_s_at	0.911837	1.002266	YIF1B
238299_at	0.910261	0.626524	
235952_at	0.907732	-0.77104	DGKH
218995_s_at	0.90641	0.765953	EDN1
224321_at	0.88726	1.37545	TMEFF2
240307_at	0.867401	0.756256	
222576_s_at	0.861625	0.781863	AGO1
242431_at	0.85313	0.661743	
212952_at	0.828935	1.395851	CTC-425F1.4
229483_at	0.813105	0.607034	
243037_at	0.807443	0.591735	
232865_at	0.806242	0.683887	AFF4
241617_x_at	0.798709	0.63713	

223185_s_at	0.787555	0.629345	BHLHE41
202648_at	0.762096	0.659284	TCF3
213826_s_at	0.758109	0.580755	H3F3A
227223_at	0.73448	0.670256	RBM39
215450_at	0.730616	0.908543	SNRPE
213736_at	0.72742	0.960418	COX5B
242110_at	0.725183	0.682279	
232264_at	0.716572	0.906203	
237006_at	0.69366	0.67368	
239655_at	0.679654	0.644959	
222413_s_at	0.654935	0.664361	KMT2C
223217_s_at	0.654782	0.861758	NFKBIZ
216246_at	0.651939	0.628325	
241775_at	0.642129	0.715711	
242121_at	0.617583	1.013166	
239597_at	0.606089	0.715479	
219858_s_at	0.60532	-0.92256	MFSD6
213249_at	0.60182	-0.5879	FBXL7
221419_s_at	0.587385	0.758604	
241425_at	0.581279	0.640169	NUPL1
238420_at	-0.58018	-1.22938	TAOK1
1560020_at	-0.58486	-2.0726	DNAJC13
241685_x_at	-0.58674	-1.50725	PURA
202549_at	-0.58808	-0.95872	VAPB
229413_s_at	-0.59025	-0.68698	
217813_s_at	-0.59166	-0.67968	SPIN1
209258_s_at	-0.59174	-0.74469	SMC3
244703_x_at	-0.59307	-0.97918	IPO9
215528_at	-0.59359	0.604277	
211612_s_at	-0.59461	-1.16222	IL13RA1
218218_at	-0.59707	-0.95986	APPL2
208042_at	-0.6	-0.89812	AGGF1
211077_s_at	-0.6051	-1.38298	TLK1
220591_s_at	-0.60596	-0.7029	EFHC2
234992_x_at	-0.61204	-0.65865	ECT2
202905_x_at	-0.61291	-0.79681	NBN
235606_at	-0.61478	-0.71715	LINC00883
219717_at	-0.61695	-0.98428	DCAF16

212220_at	-0.62179	-1.55098	PSME4
203491_s_at	-0.62212	-0.84824	CEP57
202479_s_at	-0.62698	-0.79151	TRIB2
201206_s_at	-0.62786	-0.61345	RRBP1
1561615_s_at	-0.62878	-1.52369	SLC8A1
227510_x_at	-0.62989	-0.80488	MALAT1
205369_x_at	-0.63094	-1.03863	DBT
216555_at	-0.63765	-0.6158	PRR14L
206500_s_at	-0.6384	-1.36731	MIS18BP1
217644_s_at	-0.63863	-0.88367	SOS2
212514_x_at	-0.63965	-0.87327	DDX3X
203294_s_at	-0.6398	-1.23925	LMAN1
235388_at	-0.64065	-0.61758	CHD9
232238_at	-0.64105	-1.17108	ASPM
226090_x_at	-0.64193	-1.402	RABL3
223453_s_at	-0.64622	-1.40223	ATL3
205457_at	-0.64637	-0.60744	C6orf106
208859_s_at	-0.64654	-0.78584	ATRX
218430_s_at	-0.64931	-1.43939	RFX7
209127_s_at	-0.65362	-0.68793	SART3
1558924_s_at	-0.65375	-1.30475	CLIP1
239309_at	-0.65418	-0.71975	DLX6
208624_s_at	-0.65444	-1.22076	EIF4G1
219925_at	-0.6545	-1.28282	ZMYM6
203056_s_at	-0.6567	-1.31269	PRDM2
1552378_s_at	-0.65696	-0.70115	RDH10
208097_s_at	-0.65939	-0.91118	TMX1
1557918_s_at	-0.66069	-0.85486	SLC16A1
212570_at	-0.66253	-0.59674	ENDOD1
243999_at	-0.67154	-0.74283	SLFN5
221705_s_at	-0.67348	-0.92843	SIKE1
210733_at	-0.67481	-0.84665	TRAM1
220825_s_at	-0.67535	-0.63683	KIRREL
1558527_at	-0.67879	-0.60518	PAXIP1OS
228686_at	-0.67957	-0.78508	LINC01184
221695_s_at	-0.68072	-0.8985	MAP3K2
221590_s_at	-0.68109	-0.62176	ALDH6A1
214552_s_at	-0.68219	-1.03501	RABEP1

1555594_a_at	-0.68597	-0.67191	MBNL1
207981_s_at	-0.68731	-0.63662	ESRRG
210596_at	-0.69157	-0.65964	MAGT1
239486_at	-0.69663	-1.62759	REL
214007_s_at	-0.69977	-0.95819	TWF1
211022_s_at	-0.70248	-1.39602	ATRX
201646_at	-0.70293	-0.70388	SCARB2
216069_at	-0.70533	-0.81607	PRMT2
212092_at	-0.70544	-1.06395	PEG10
207924_x_at	-0.70634	-0.81621	PAX8
215992_s_at	-0.70644	-1.26591	RAPGEF2
241091_at	-0.7065	-0.92343	AGO3
202601_s_at	-0.70738	-0.99172	HTATSF1
207326_at	-0.71171	-0.78921	BTC
222456_s_at	-0.7118	-1.44523	LIMA1
228497_at	-0.71278	-0.62809	SLC22A15
1555106_a_at	-0.71861	-1.04745	CTDSPL2
1557100_s_at	-0.71901	-0.88293	HECTD1
242468_at	-0.71984	0.614569	
204909_at	-0.72609	-1.34299	DDX6
219234_x_at	-0.72844	-0.96585	SCRN3
228530_at	-0.73712	-0.61734	MZT1
1558143_a_at	-0.73716	-0.65399	BCL2L11
211814_s_at	-0.74639	-0.8622	CCNE2
1554390_s_at	-0.74881	-1.09034	ACTR2
213446_s_at	-0.74884	-1.08525	IQGAP1
1558015_s_at	-0.75465	-1.08808	ACTR2
201101_s_at	-0.7548	-0.97688	BCLAF1
208930_s_at	-0.7576	-0.95641	ILF3
222508_s_at	-0.75818	-1.31456	ARGLU1
241752_at	-0.76142	-1.10609	SLC8A1
220946_s_at	-0.76254	-0.97605	SETD2
239148_at	-0.76373	-0.71258	MARVELD3
223584_s_at	-0.76378	-1.56481	KBTBD2
242475_at	-0.76476	-0.90236	
207213_s_at	-0.76623	-0.88722	USP2
222586_s_at	-0.76922	-0.69516	OSBPL11
224775_at	-0.77035	-0.76699	IWS1

217576_x_at	-0.77053	-0.73725	SOS2
238959_at	-0.77152	-0.61245	LARP4
1554588_a_at	-0.77292	-0.76592	TTC30B
222439_s_at	-0.77405	-0.72471	THRAP3
244396_at	-0.77718	-1.78543	G3BP1
237444_at	-0.77775	-1.09563	KIF13A
1556551_s_at	-0.78132	-1.24222	SLC39A6
219558_at	-0.78406	-1.13561	ATP13A3
242630_at	-0.78581	-0.61965	
204680_s_at	-0.78861	-0.58446	RAPGEF5
243857_at	-0.78867	-0.89082	MORF4L2
203990_s_at	-0.7903	-1.07345	KDM6A
206652_at	-0.79368	-0.97931	ZMYM5
221985_at	-0.79552	-0.96292	KLHL24
223940_x_at	-0.79977	-0.93532	MALAT1
235461_at	-0.80232	-0.81021	TET2
205787_x_at	-0.80429	-0.61296	LOC441155
228186_s_at	-0.80465	-0.6411	RSPO3
217208_s_at	-0.81394	-0.97579	DLG1
223882_at	-0.81489	-1.52357	FAM172A
230926_s_at	-0.82838	-1.15984	ODF2L
227276_at	-0.8289	-0.822	PLXDC2
229281_at	-0.83447	-0.85073	NPAS3
215092_s_at	-0.83466	-1.20503	NFAT5
1553304_at	-0.83608	-1.10991	LSM14B
1555058_a_at	-0.83633	-0.6626	LPGAT1
231174_s_at	-0.84052	-1.26928	
223888_s_at	-0.84543	-1.66618	LARS
235050_at	-0.84817	-0.66527	SLC2A12
212113_at	-0.84868	-0.68526	ATXN7L3B
200956_s_at	-0.85071	-0.86448	SSRP1
220885_s_at	-0.8555	-1.39354	CENPJ
201562_s_at	-0.86729	-1.03174	SORD
217599_s_at	-0.86734	-2.01953	MDFIC
212065_s_at	-0.87208	-1.04315	USP34
1569472_s_at	-0.87461	-1.27069	TTC3
203293_s_at	-0.88144	-1.23682	LMAN1
200727_s_at	-0.89101	-1.17222	ACTR2

235030_at	-0.89301	-1.2157	NXPE3
226533_at	-0.89306	-1.15708	HINT3
216521_s_at	-0.91898	-1.12833	BRCC3
241403_at	-0.9212	-1.41339	CLK4
202118_s_at	-0.94325	-1.11263	CPNE3
214869_x_at	-0.95567	-0.95822	GAPVD1
203899_s_at	-0.95712	-0.95581	CRCP
223925_s_at	-0.96659	-1.26551	MTPN
227741_at	-0.96705	-0.94998	PTPLB
1558093_s_at	-0.96978	-1.10479	MATR3
243552_at	-0.97826	-1.10792	MBTD1
238815_at	-0.98174	-0.66659	LRRTM1
228986_at	-0.99485	-1.43157	OSBPL8
218457_s_at	-0.99542	-0.90331	DNMT3A
1557128_at	-1.00174	-0.75351	FAM111B
221986_s_at	-1.01098	-1.33873	KLHL24
219387_at	-1.01737	-1.37569	CCDC88A
235722_at	-1.02005	-1.60816	SYNJ2BP
211947_s_at	-1.03594	-1.43942	PRRC2C
1557227_s_at	-1.04962	-2.18135	TPR
212468_at	-1.06265	-1.43139	SPAG9
210544_s_at	-1.0783	-1.06058	ALDH3A2
212384_at	-1.08681	-1.456	DDX39B
229346_at	-1.09302	-1.15171	NES
235653_s_at	-1.11473	-1.045	THAP6
217620_s_at	-1.11767	-1.39267	PIK3CB
214374_s_at	-1.15931	-2.0827	PPFIBP1
236957_at	-1.16897	-1.66782	CDCA2
224568_x_at	-1.17995	-1.03892	MALAT1
212650_at	-1.18529	-1.44017	EHBP1
212720_at	-1.18643	-1.57027	PAPOLA
1553106_at	-1.19821	-2.01813	C5orf24
238013_at	-1.20596	-1.82384	PLEKHA2
212105_s_at	-1.21259	-1.52794	DHX9
1557129_a_at	-1.25085	-2.22678	FAM111B
201708_s_at	-1.27311	-1.13997	NIPSNAP1
1565867_a_at	-1.31482	-1.21865	ZC3H11A
1558378_a_at	-1.32556	-1.50186	AHNAK2

233819_s_at	-1.3652	-2.41524	LTN1
227454_at	-1.36629	-0.65263	TAOK1
211081_s_at	-1.39046	-1.82853	MAP4K5
209344_at	-1.40596	-2.00066	TPM4
207754_at	-1.42327	-1.34497	RASSF8
235645_at	-1.42847	-2.06794	ESCO1
205018_s_at	-1.44471	-2.06373	MBNL2
229399_at	-1.45396	-2.07194	CCDC186
233827_s_at	-1.45407	-2.15614	SUPT16H
1555436_a_at	-1.46473	-1.43647	AFF4
241683_at	-1.47526	-1.82988	HECTD1
228919_at	-1.48149	-0.98284	
212107_s_at	-1.48337	-1.5442	DHX9
1555559_s_at	-1.51004	-0.95708	USP25
235852_at	-1.56164	-1.51306	STON2
214786_at	-1.56338	-1.34048	MAP3K1
208297_s_at	-1.5659	-1.64766	EVI5
230180_at	-1.60518	-1.30448	DDX17
1554029_a_at	-1.61076	-1.80102	TTC37
1553122_s_at	-1.62838	-2.6051	RBAK
221220_s_at	-1.68394	-2.28557	SCYL2
242918_at	-1.73237	-1.17096	NASP
235588_at	-1.77313	-1.98522	ESCO2
1560017_at	-2.03177	-2.71891	TMTC3
217878_s_at	-2.13803	-2.39131	CDC27
235216_at	-2.22044	-2.60938	ESCO1
213286_at	-2.35232	-2.31648	ZFR
33148_at	-2.48151	-2.79576	ZFR

Supplementary Table 3. List of consensus upstream regulators that are computationally predicted to be activated or inhibited after paclitaxel treatment in KURAMOCHI and OVSAHO cells.

KURAMOCHI Upstream Regulators	Activation z-score	p-value of overlap	OVSAHO Upstream Regulators	Activation z-score	p-value of overlap
bicuculline	2.748	3.42E-03	bicuculline	-2	1.00E+00
camptothecin	3.571	2.94E-09	camptothecin	2.028	2.92E-04
CD44	2.462	2.68E-05	CD44	-2.727	1.00E+00
CSF2	4.105	4.46E-08	CSF2	-2.385	4.06E-01
ESR1	-2.831	1.22E-17	ESR1	-4.16	1.68E-12
etoposide	2.311	3.18E-05	etoposide	-2.082	2.65E-01
fenofibrate	-2.837	1.42E-02	fenofibrate	-3.712	2.64E-01
gentamicin	3.104	1.47E-03	gentamicin	-2.208	1.00E+00
HGF	4.984	3.47E-15	HGF	-3.933	5.15E-03
hyaluronic acid	2.262	8.35E-08	hyaluronic acid	-2.196	8.67E-02
IL1	5.034	1.78E-06	IL1	2.54	1.61E-01
IL18	3.757	9.87E-04	IL18	2.393	1.00E+00
KLF3	2.09	3.74E-01	KLF3	2.813	1.15E-01
Map3k7	2.98	3.74E-03	Map3k7	2.111	3.01E-02
NUPR1	3.81	3.01E-04	NUPR1	2.25	7.47E-02
Pdgf (complex)	3.469	1.48E-04	Pdgf (complex)	-2.033	1.10E-01
PP2/AG1879	-3.211	1.26E-04	PP2/AG1879	-2.045	1.70E-01
TLR9	2.855	1.82E-03	TLR9	2.056	4.13E-01

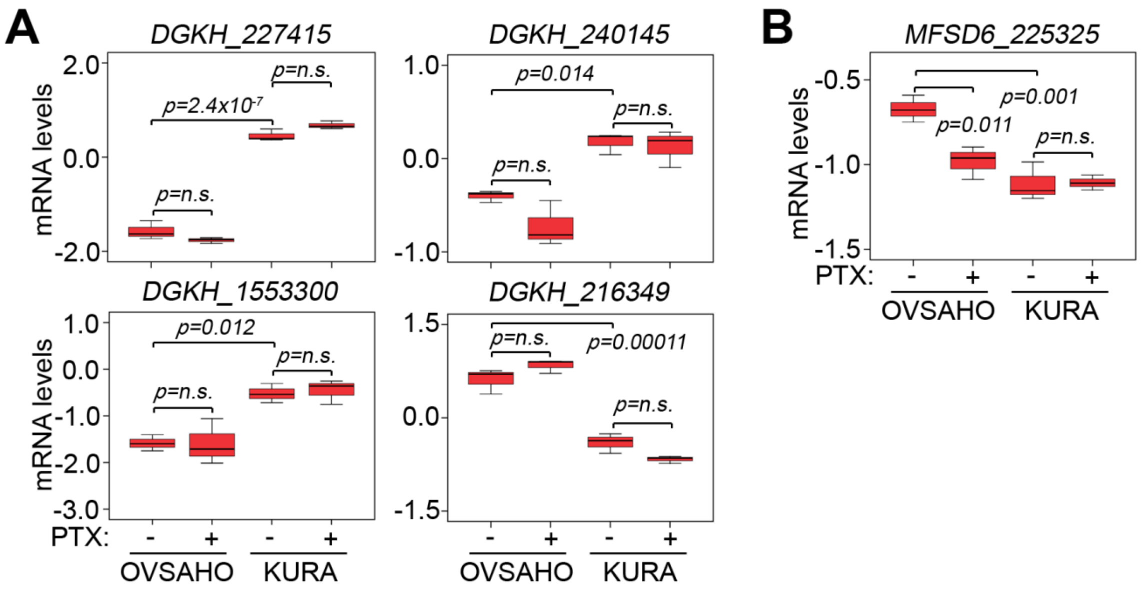

Supplementary Figure 1. Transcriptional profiling of *DGKH* and *MFSD6* with other probe identifiers in microarray analysis against OVSAHO and KURAMOCHI cells post-treatment without or with paclitaxel. (A) *DGKH* mRNA levels detected by different probes 227145, 240145, 1553300 and 216349 in microarray dataset (GSE58032) against OVSAHO and KURAMOCHI cells post-treatment without or with paclitaxel at the concentration of 10 x IC_{50} for 24 hours. (B) *MFSD6* mRNA levels detected by probe 225325 in the same condition as above. Data from three independent experiments were shown in median ± SD. The statistical differences were analyzed by One-way ANOVA using Turkey's test.

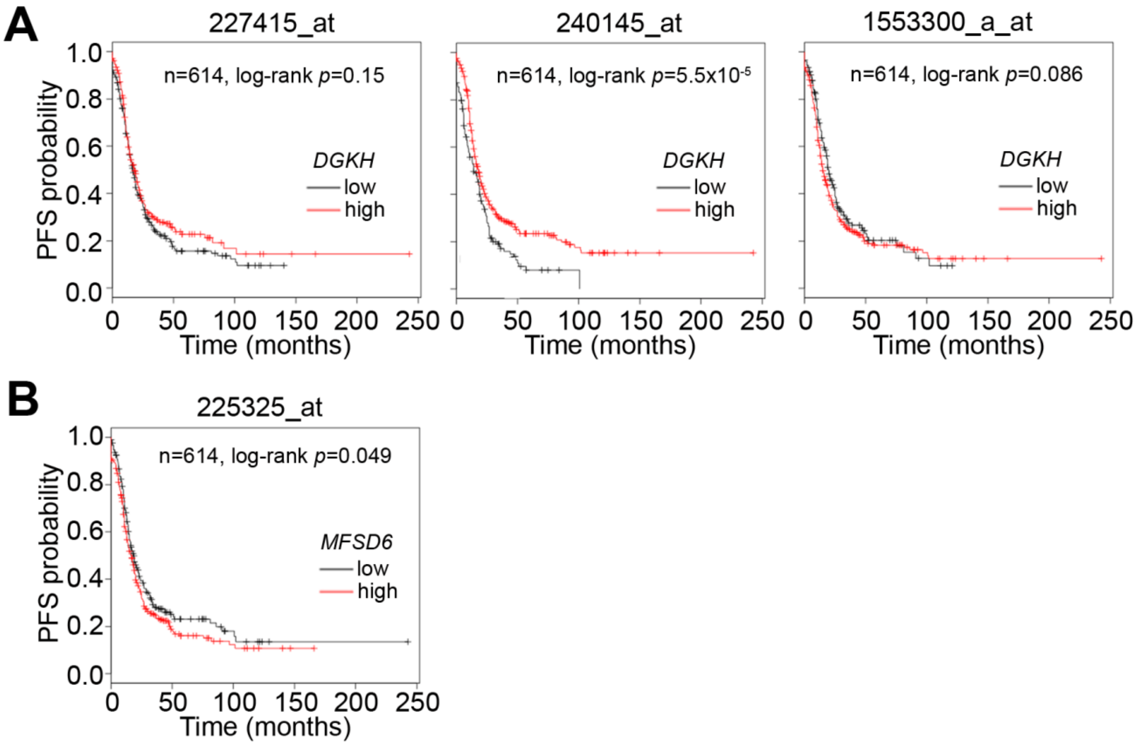

Supplementary Figure 2. Prognostic significance of different *DGKH* and *MFSD6* probes in K-M Plotter database against ovarian cancer patients. (A and B) Prognostic estimation of *DGKH* probes 227145, 240145 and 1553300 (A) and *MFSD6* probe 225325 (B) towards the survival rates of breast cancer patients under the condition of progression-free survival (RFS) probability by using K-M Plotter database.

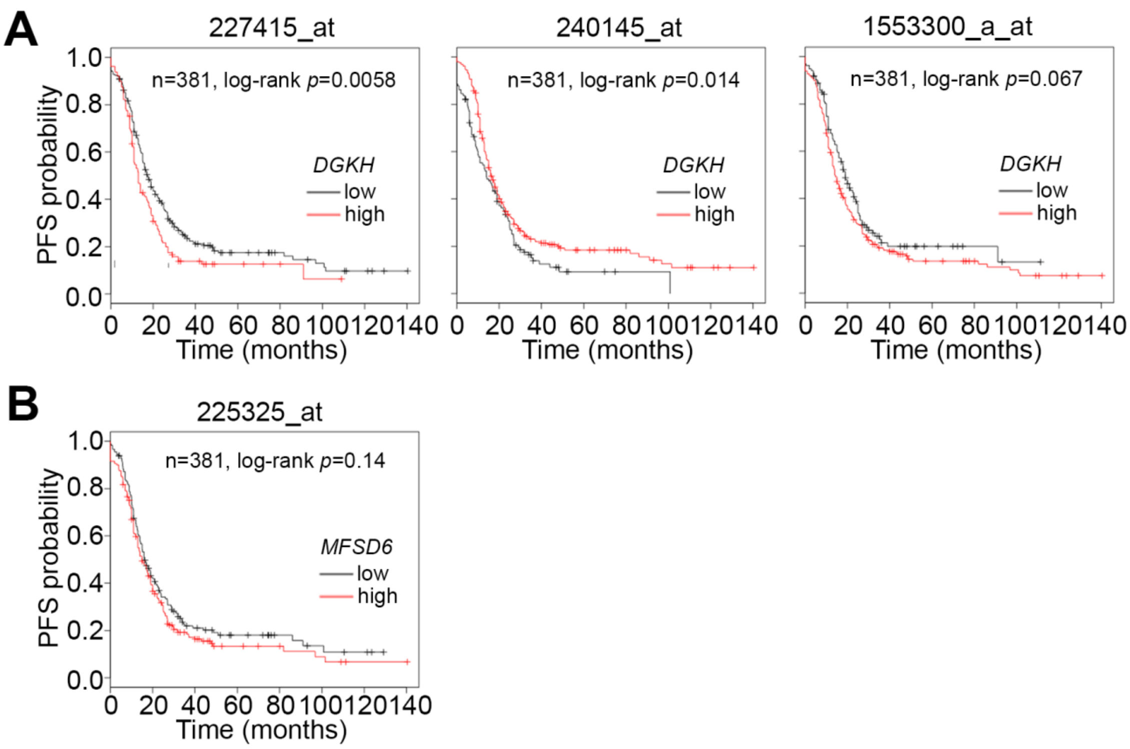

Supplementary Figure 3. Prognostic significance of different *DGKH* and *MFSD6* probes in K-M Plotter database against ovarian cancer patients who received post-operative taxol-based chemotherapy. (A and B) Prognostic estimation of *DGKH* probes 227145, 240145 and 1553300 (A) and *MFSD6* probe 225325 (B) towards the survival rates of breast cancer patients with taxol treatment under the condition of progression-free survival (RFS) probability by using K-M Plotter database.

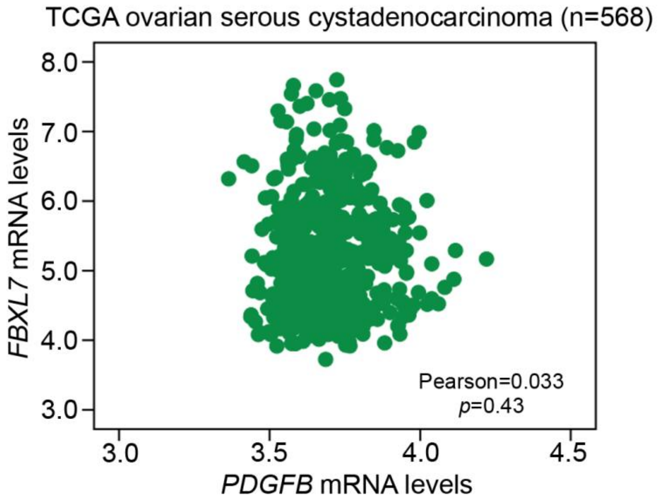

Supplementary Figure 4. Correlation between *FBXL7* and *PDGFB* mRNA levels in tissues derived from patients with ovarian serous cystadenocarcinoma using TCGA database. The statistical significance of correlations was analyzed by using Pearson's test.
